# Mixed venous versus central venous oxygen saturation in patients undergoing on pump beating coronary artery bypass grafting

**DOI:** 10.4103/1658-354X.65129

**Published:** 2010

**Authors:** Ahmad Alshaer, Mohamed Essam Abdel-Meguid, Osama Ibraheim, Khaled Fawzi, Ibrahim AbdulSalam, Saad Sheta, Khaled M. Abdullah, Ahmed El-Demerdash, Raed Al-Satli, Mohamed AbdelAll, Bakir M. Bakir, Nezar AlNahal, Yasser Abdulrahman, Hanaa AlHamoud

**Affiliations:** *Cardiac Anesthesia Division, King Fahad Cardiac Center, Riyadh, Saudi Arabia*; 1 *Consultant Cardiac Anaesthesia, King Fahad Military Medical Complex, Dhahran, Saudi Arabia*; 2 *Department of Anaesthesia, King Khalid University Hospital, King Saud University, Riyadh, Saudi Arabia*; 3 *Anesthesia Department, King Fahad Military Medical Complex, Dhahran, Saudi Arabia*; 4 *Anaesthesia Department, Ain Shams University, Cairo, Egypt*; 5 *Consultant Cardiac Anaesthesia, King Fahad National Guard Hospital, Riyadh, Saudi Arabia*; 6 *Cardiac Surgery Division, King Fahad Cardiac Center, King Saud University, Riyadh, Saudi Arabia*; 7 *Department of Surgery, King Saud University, Riyadh, Saudi Arabia*

**Keywords:** *Coronary artery bypass grafting*, *mixed venous oxygen saturation*, *coronary artery bypass grafting*, *mixed venous oxygen saturation*, *central venous oxygen saturation*

## Abstract

**Objective:**

To examine the validity of central venous oxygen saturation (ScvO_2_) as a numerical substitution of mixed venous oxygen saturation (SvO_2_) in adult patients undergoing normothermic on pump beating coronary artery bypass grafting (CABG).

**Materials and Methods:**

Prospective clinical observational study was done at King Khalid University Hospital, King Saud University, Riyadh, Kingdom of Saudi Arabia. Thirty four adult patients scheduled for coronary artery surgery were included. Patients were monitored by a pulmonary artery catheter (PAC) as a part of our routine intraoperative monitoring. SvO_2_ and ScvO_2_ were simultaneously measured 15 minutes (*T*1) and 30 minutes (*T*2) after induction of anesthesia, 15 and 30 minutes after initiation of cardiopulmonary bypass (*T*3 and *T*4), and 15 and 30 minutes after admission to intensive care unit (*T*5 and *T*6).

**Results:**

ScvO_2_ showed higher reading than SvO_2_ all through our study. Our results showed perfect positive statistically significant correlation between SvO_2_ and ScvO_2_ at all data points. Individual mean of difference (MOD) between both the readings at study time showed MOD of 1.34 and 1.44 at *T*1 and *T*2 simultaneously. This MOD was statistically insignificant, but after on pump beating normothermic bypass was initiated; MOD was 5.2 and 4.4 at *T*3 and *T*4 with high statistical significance. In ICU, MOD continues to have high statistical significance, MOD was 6.3 at *T*5 and at *T*6 it was 4.6.

**Conclusions:**

In on pump beating CABG patients; ScvO_2_ and SvO_2_ are not interchangeable numerically. ScvO_2_ is useful in the meaning of trend; our data suggest that ScvO_2_ is equivalent to SvO_2_ , only in the course of clinical decisions as long as absolute values are not required.

## INTRODUCTION

Mixed venous oxygen saturation (SvO_2_) is a valuable measurement in hemodynamically unstable patients during cardiac surgery.[1,2] SvO_2_ has been used to assess to what extent the cardiopulmonary system meets the metabolic demands of the various tissues and to provide an index of tissue oxygenation.[3] Furthermore, it allows calculation of tissue oxygen consumption, oxygen extraction ratio, and the degree of pulmonary venous admixture.[4] However, SvO_2_ measurement is obtained only from a correctly positioned pulmonary artery catheter (PAC).

Significant complications are associated with the use of a PAC.[5,6] As such, central venous oxygen saturation (ScvO_2_) represents an attractive alternative to SvO_2_ because central venous catheterization is easier and less invasive than pulmonary artery (PA) catheterization.[7]

The clinical applicability of substituting ScvO_2_ for SvO_2_ in different clinical situations is still not fully studied. Open heart surgery is a unique clinical situation where there is a great variation during the surgery in hemodynamic and filling indices.

In a previous study,[8] we aimed to examine the correlation between ScvO_2_ sampled from a standalone central venous line (CVL) and SvO_2_ sampled from PAC port, to test the validity of the clinical applicability of substituting ScvO_2_ from CVL for SvO_2_ in adult patients with poor myocardial function undergoing open heart coronary artery bypass grafting surgery (CABG).[8] In the current study, we are testing the same hypothesis in patients with normal functioning myocardium undergoing CABG using the on pump beating technique, while measuring the ScvO_2_ from a proximal PAC port.

## MATERIALS AND METHODS

After obtaining approval from the Institutional Review Board at the College of Medicine, King Saud University, Riyadh, KSA, and informed consent from each participant, we studied 34 patients scheduled to undergo CABG using on pump beating normothermic cardiopulmonary bypass.

The present study is a prospective observational study. Thirty four adult patients of either sex, aged above 40 years, suffering from coronary heart disease with normal myocardial function, scheduled for elective CABG surgery were included in the study.

A standardized balanced anesthetic technique was used for all the patients; patients were premedicated with lorazepam 2 mg orally at the night of surgery and morphine 0.1 mg/kg IM preoperatively. On receiving the patient in operating room, standard monitoring was instituted. Peripheral venous as well as radial artery cannulae were inserted. Induction is with sufentanil 1-1.5 μg/kg, midazolam 0.05-0.1 mg/kg, and rocuronium 0.9 mg/kg, then a maintenance infusion of the same induction agents, that is, sufentanil 0.2 μg/kg/h, midazolam 1.5 μg/kg/h and rocuronium 0.5 mg/kg/h supplemented with sevoflurane was given as required. Induction doses as well as anesthetic maintenance supplementation were guided by BIS^®^ monitoring (Aspect *medical Systems, inc*, Norwood, Massachusetts, USA ), and signs of lack of analgesia correlated with hemodynamic changes were managed as appropriate. A PAC was inserted after induction of anesthesia enabling monitoring of SvO_2_, ScvO_2_ as well as other derived parameters. The lungs were mechanically ventilated with a tidal volume of 8 mL/kg and FiO_2_ of 0.4 oxygen in air mixture, while ventilatory rate was adjusted to maintain a PaCO_2_ of 32–36 mmHg.

A 7.5F PAC (Edwards Lifesciences; Irvine, CA, USA) that was 110 cm in length and had the right atrial lumen positioned 30 cm from the tip was inserted through the internal jugular vein using a percutaneous 8.5F sheath introducer (Edwards Lifesciences). A pressure tracing obtained from the proximal PAC port was used to ascertain correct positioning in the right atrium (RA). Postoperative portable chest radiograph and the presence of PA pressure tracings confirmed the location of the distal port in the PA.

Immediately after the insertion of the PAC, each patient had one set of paired blood samples drawn in random order simultaneously from the distal and proximal ports of PAC. The first 2 mL blood drawn for each sample was discarded to prevent contamination with flushing fluid. Blood was sampled from distal PAC port while the catheter balloon deflated. We then measured the pulmonary artery occlusive pressure (PAOP) and cardiac output (CO) by the thermo dilution method as well as other hemodynamic calculations.

Previous data were collected 15 and 30 minutes after induction of anesthesia (*T*1, *T*2), 15 and 30 minutes after initiation of cardiopulmonary bypass (*T*3, *T*4) and 15 and 30 minutes postadmission to intensive care unit (*T*5 and *T*6).

Blood samples were drawn simultaneously from the PA and RA at six different data points mentioned. A standard volume of 1 mL blood was obtained from each site, and oxygen saturations per blood sample were determined using the blood gas analyzer (QS 50® ; Radiometer, Copenhagen, Denmark).

All surgeries were done by the same surgeon, using the on pump beating normothermic cardiopulmonary bypass.

### Data analysis

Data were analyzed using statistical software package (Graph Pad In Stat® version 3.00 for Windows, Graph Pad Software Inc., San Diego, CA, USA) and presented as numbers, mean (standard deviation [SD]), or ratio. Data were compared using the parametric or the nonparametric versions of analysis of variance (ANOVA) followed by the appropriate *post hoc* analysis if significance was detected. *P* values < 0.05 were considered significant.

Demographic and hemodynamic data were compared using the Student‘s *t*-test with levels of significance adjusted according to the method of Bonferroni for multiple comparisons. *P* < 0.05 is deemed to denote a significant difference.

The correlation between SvO_2_ and ScvO_2_ was evaluated by linear regression analysis and Pearson test followed by the *F* test. Mean of difference (MOD) between simultaneously measured SvO_2_ and ScvO_2_ individual values were calculated. The Student‘s *t*-test was used to determine whether the mean difference was significantly different from zero.

## RESULTS

Patients‘ demographic and operative data are shown inTable 1. The measured hemodynamic parameters and hemoglobin concentration values are listed inTable 2. Other parameters assessed such as Cardiac Index, PAOP and CVP were not applicable during bypass (*T*3 and *T*4).

**Table 1:** Demographic and operative data

Number of patients	34
Age (years)	57.1 ± 5.2
Sex M/F (n)	25/9
Preoperative Hb% (g/dL)	12.1 ± 1.57
No. of grafts	2.9 ± 0.81
LVEF(%)	47.41 ± 5.92
CPB time (minutes)	109.4 ± 18.51

Data are expressed as mean ± SD.

**Table 2:** Hemodynamic parameters and hemoglobin concentrations at the different data points

	*T*1	*T*2	*T*3	*T*4	*T*5	*T*6	*P* value
SaO_2_ (%)	99.21 ± 1.23	99.34 ± 0.81	99.49 ± 0.92	99.59 ± 0.72	99.3 ± 0.94	99.21 ± 0.69	NS
CI (L/min/M^2^)	3.32 ± 1.22	3.13 ± 1.14	NA	NA	4.32 ± 0.89	3.89 ± 0.81	NA
PAOP (mmHg)	17.12 ± 5.93	18.32 ± 5.16	NA	NA	13.33 ± 5.9	14.13 ± 5.44	NA
CVP (mmHg)	14.23 ± 8.15	11.62 ± 6.26	NA	NA	13.96 ± 7.34	12.93 ± 8.52	NA
Hb (g/dL)	12.41 ± 1.7	12.1 ± 1.42	10.1 ± 2.32	8.98 ± 2.56	9.67 ± 1.32	10.2 ± 3.44	<0.001

Data are expressed as mean ± SD, significant (*P* < 0.05) NA, not applicable; NS, not significant.

ScvO_2_ showed higher values than SvO_2_ all through our study [Table 3]. Data showed perfect positive statistically significant correlation between SvO_2_ and ScvO_2_ at all study times, individual MOD between both the readings at study time showed MOD of 1.34 and 1.44 at *T*1 and *T*2 simultaneously, this MOD was statistically insignificant; but after bypass was initiated, MOD was 5.2 and 4.4 at *T*3 and *T*4 with high statistical significance; after bypass MOD continues to have high statistical significance, it was 6.3 at *T*5 and at *T*6 it was 4.6 [Table 3].

**Table 3:** Correlation between SvO_2_ and ScvO_2_ at the different data points

	SvO_2_	ScvO_2_	MOD	*P* value (t test)	Correlation coefficient (*r*)	*P* (*F* test)
*T*1	80.98 ± 5.26	82.32 ± 6.035	1.34	0.2140	0.752	0.001
*T*2	82.93 ± 5.17	81.5 ± 3.47	1.43	0.1760	0.7914	<0.0001
*T*3	78.1 ± 6.31	83.3 ± 5.31	5.2	<0.0005	0.6301	0.0099
*T*4	80.8 ± 6.1	85.2 ± 4.93	4.4	0.0017	0.687	0.0009
*T*5	79.12 ± 5.91	85.42 ± 6.3	6.3	<0.001	0.7901	0.0001
*T*6	76.7 ± 5.96	81.3 ± 5.67	4.6	0.0018	0.6901	0.0117

Data are expressed as mean ± SD, significant (*P* < 0.05).

## DISCUSSION

In the present study, blood was taken from the RA to be representative of whole body central venous blood. Presumably, this position placed the ScvO_2_ sampling site sufficiently distal into the RA to allow for the mixing of blood from the superior and inferior venacavae.

Results showed a lower value of SvO_2_ compared to ScvO_2_, a possible explanation for the decrease in SO_2_ from ScvO_2_ to SvO_2_ is the myocardial extraction of O_2_ as blood flows through the right ventricle into the PA. Although, to our knowledge, the rate of O_2_ diffusion from ventricular blood into the myocardium has not been quantified, we consider this possibility unlikely. A more likely hypothesis is that atrial blood, as it moves toward the PA, mixes with blood of lower O_2_ content. It is also possible that decrease in SvO_2_ resulted from blood mixing with blood draining from coronary sinus in RA and the Thebesian veins in the right ventricle.[10]

Experimental studies in animals showed an excellent correlation between ScvO_2_ and SvO_2_ . Reinhart *et al*.[11] found a Spearman correlation coefficient of 0.97 in anesthetized dogs over a broad range of cardio-respiratory conditions, including hypoxia, hemorrhage, and resuscitation. Schou *et al*.[12] also found a correlation coefficient of 0.97 between ScvO_2_ and SvO_2_ in pigs that had been subjected to conditions of graded hypoxemia. Of note, both studies found SvO_2_ to be consistently lower than ScvO_2_.

**Figure 1:**
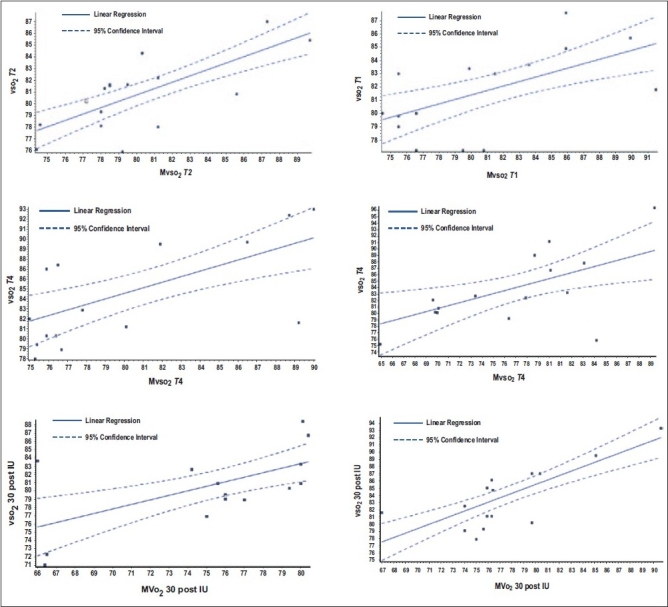
The correlation between SvOm_2_ and ScvO_2_ in all data points

Our data showed perfect positive statistically significant correlation between SvO_2_ and ScvO_2_ at all data points [Table 3 and Figure 1 a–f].

In the present study, individual mean of difference between both the readings at data points showed MOD of 1.34 and 1.44 at *T*1 and *T*2 simultaneously. This MOD was statistically insignificant, meaning that they are interchangeable numerically; but after bypass was initiated, MOD was 5.2 and 4.4 at *T*3 and *T*4 with high statistical significance. In the ICU; MOD continues to have high statistical significance, MOD was 6.3 at *T*5 and 4.6 at *T*6. The poor agreement between the values of SvO_2_ and ScvO_2_ after initiation of cardiopulmonary bypass presented here may be secondary to the acute changes in hemodynamics accompanying the shift to CPB with hemodilution and the nonpulsatile flow pattern. Moreover, catheter position might be altered while cannulating the RA for bypass and by myocardial manipulation during surgery. This agrees with other studies[13,14] comparing measures of ScvO_2_ and SvO_2_ in critically ill patients. Similarly, poor agreement results appeared with other studies comparing SvO_2_ and ScvO_2_ in hemodynamically unstable patients. These studies were performed outside the context of cardiac surgery, with heterogeneous groups of patients in septic,[15] cardiogenic,[16] and neurogenic shock,[17] and all of them reported a poor agreement between SvO_2_ and ScvO_2_ individual values. Schmitz *et al*.[17] showed also that patients with normal cardiac index values, ScvO_2_, could not be substituted for SvO_2_ after cardiac surgery with cardiopulmonary bypass.

In our previous study,[8] we found the same pattern of oxygen saturation reduction while the blood moves from RA to the PA and a positive correlation between the readings obtained from the measured samples, but we could not find relation between RA and PA data.

### Limitations of the study

In spite of the care taken to have the PAC positioned accurately, its position is bound to be altered by the insertion of atrial cannulae for bypass, and by cardiac mobilization during surgery.

## CONCLUSIONS

In CABG patients; ScvO_2_ and SvO_2_ are not interchangeable numerically as they have failed to keep insignificant MOD when normothermic CPB was used, but had strong positive and significant correlations throughout the operative course. This makes ScvO_2_ useful in the meaning of trend; these data suggest that ScvO_2_ is equivalent to SvO_2_ in the course of clinical decisions as long as absolute values are not required.
